# Storytelling, behavior planning, and language evolution in context

**DOI:** 10.3389/fpsyg.2014.01131

**Published:** 2014-10-15

**Authors:** Glen McBride

**Affiliations:** School of Psychology, University of QueenslandBrisbane, QLD, Australia

**Keywords:** behavior generation, evolution, experiences, gestures, language, miming, mirror neurons, storytelling

## Abstract

An attempt is made to specify the structure of the hominin bands that began steps to language. Storytelling could evolve without need for language yet be strongly subject to natural selection and could provide a major feedback process in evolving language. A storytelling model is examined, including its effects on the evolution of consciousness and the possible timing of language evolution. Behavior planning is presented as a model of language evolution from storytelling. The behavior programming mechanism in both directions provide a model of creating and understanding behavior and language. Culture began with societies, then family evolution, family life in troops, but storytelling created a culture of experiences, a final step in the long process of achieving experienced adults by natural selection. Most language evolution occurred in conversations where evolving non-verbal feedback ensured mutual agreements on understanding. Natural language evolved in conversations with feedback providing understanding of changes.

## BACKGROUND

In 1990, Behavioral and Brain Sciences published perhaps the most comprehensive examination of the evolution of natural languages. Professors Pinker and Bloom (1990) led the discussion. The discussion stimulated further contributions to the topic. Corballis (2003) presented an excellent history of accounts of a gestural origin of language as well as strong arguments for such an origin. In 2009, he presented a summary of the progress since the 1990 discussion. Tomasello (2010) presented a different account of gestural origins that included a new form of gesture, pantomimes, which extend the semantic content of gestures, within the capabilities of a hominin but beyond those of apes. His other advance, developed strongly in (2014) was the case for shared mutual understanding and intentionality, a common ground in interactions which he shows as essential even for pointing gestures in hominins. Corballis (2003) and Tomasello (2010) made clear that the calls of primates are fixed and unlikely to contribute to the origins of human language, so that a gestural origin is more likely. Neither writer showed why gestures might lead to increasing numbers of descendants, emphasized as essential in 1990. It is as though the reproductive advantages of a gestural language from the beginning are obvious.

The need for Tomasello’s (2010) common ground in interactions is clear, but it is difficult to see two adult primates who have had a long social relationship involving many interactions, coming together, alerting as one enters what Hediger (1962) called the personal space of the other, without both already knowing what will happen next and the probable outcome of the interaction, a shared understanding and intentionality, a common ground. Social relationships in many gregarious species would build such common grounds. Creating common grounds is probably part of all mammalian and avian social relationships, perhaps often only “simple” mainly agonistic ones. Forming shared common ground, limited or general, positive or negative, is central to forming relationships.

## HOMININ SOCIETIES

Understanding the hominin societies in which the first steps to language occurred is important. We assume the hominins were bipedal. We also know that somewhere on the hominin pathway the primate troops changed from multi-male, multi-female troops to bands of pair families. Reasons for the transitions can only be speculative but the two transitions were relevant to language evolution. I suggest they were related.

Becoming bipedal must have been a very stressful period in evolution. Short periods of bipedal walking were always an option for terrestrial primates. More important is why in one band, individuals found it essential to remain erect for long periods, setting conditions for natural selection to begin the transition to bipedalism. McBride (2000) suggested that the first step was the ability of young males to defeat older males for rank with sharp sticks. Animals winning high rank with sticks could not readily discard them. Retaining sticks soon gave other uses for them; they could also be used by both genders for digging while foraging.

Wheeler (1988, 1991) suggested that bipedalism and loss of hair arose to reduce the heat load on apes moving into open unshaded country. Erect females would always be initially handicapped by this posture but lack of shade has not brought any other mammal to lose hair. The individuals had to decide whether the heat load justified the standing erect with the huge pain load involved in standing/walking for long periods.

We can only speculate on why these changes occurred. Yet it could only have been an intragroup demand that pressed on individuals all and every day; the transition was painful. We know that already in *Ardipithecus* (White et al., 2009), perhaps the earliest erect hominin, the great canines, fierce threats/weapons for millions of years were already reduced with minimum sex dimorphism. A replacement weapon would have made them irrelevant almost immediately.

The role of weapons in the transition becomes relevant because of the competitiveness of the chimpanzee type troops as an ancestral hominin model, (Tomasello, 2010, 2014). Armed hominins had to become more cooperative, cautious with weapons that could easily wound each other especially with/by youngsters.

Standing erect for long periods must have been painful. Youngsters could manage better than adults. All would have needed to rest, leaning on their sticks and often finding opportunities to sit. Often adults would have reverted to quadruped walking, dragging sticks along the ground.

Males had always walked erect occasionally, taking turns in long grass to avoid blundering into hidden predators. For the females, the bipedal step was more stressful. They were always pregnant or carrying offspring, perfectly adapted to an ancient horizontal spine. A vertical spine changed the suspension of every internal organ, changed the demands of heavy pregnancy and carrying infants, while revising the whole behavior of infant carrying and nursing; adult females were obviously last to achieve full bipedalism. They needed help.

Both bonobo and our ancestor females made the change to “anytime sex.” It was as though they came into their first estrus with an interest in sex and lost the ability to turn off the estrus after ovulation. In bonobos, the change in society gave control of access to sex into the hands of the females and it has remained there; they needed no help. Powerful males no longer controlled mating. In the human ancestral line, I suggest anytime sex arrived while changing to bipedalism. The formation of consort pairs, common in chimpanzees, was then welcome, with dominant males prepared to protect and support sexually active females, even to sharing food. The support was not every three or four years during estrus but daily throughout life; the price was anytime sex, now privately as in chimpanzee consort pairs. As anytime sex females reached maturity, they would immediately be taken by males powerful enough to protect them. Some young females reaching maturity may have still moved between troops seeking consort males. With daily sex, advertising ovulation became irrelevant and disruptive to pairs seeking separation. Ovulation became and remains essentially silent. The vulva became less conspicuous when standing erect and selection completed the elimination of what was now a disruptive estrus display. Males remained in control of females and mating in consort pairs.

Diamond (1991, pp. 67) listed six models for the evolution of silent ovulation; none involved the established consort pair model already well known in chimpanzees.

Hrdy (2009) suggests that the extra help erect and presumably unbonded females have always needed with offspring came from many sources, from mating with several males, all of whom might feel prepared to help, or from close kin, older offspring, grandparents, or siblings. Kin may well be involved, but we know the transition to mainly pair matings occurred. She did not consider consort pairs models beginning with males helping and defending sexually available females, separately within troops. Pairs do not mean absence of other females helping. Models can only be speculative.

We know in time, troops changed, probably from the typical chimpanzee structure to an erect pair family societal structure in bands of hominins. The troops would remain foragers and scavengers. They could no longer run competently, but could face predators with a wall of sharp sticks, probably eventually learning to scavenge, driving predators from their prey.

Presumably troops remained territorial; all descendants have retained suspicion/hostility toward neighboring bands. Pairs were the key to preparing these hominins for better communication. Pairs were always together, cooperating, foraging, and sharing with their young and each other. They rested and nested together. Other pair families were always nearby; neighboring females were probably friendly, groomed each other and were sometimes relatives. There had always been social relationships in mammals, well developed in primates. The constant need for cooperation within the pair family ensured that communication was developing in 100s of small ways, by gesture, sounds, and facial expression. Tomasello (2010) showed how a touch on mother’s back became a signal for mother to stop, crouch for the offspring to climb onto her back. Living closely together fosters such communicative developments. Families nearby understood each other’s communications; they became a culture of the troop (Tomasello, 2010), carried between troops by young females seeking mates. Gestures are part of the lives of apes and people, especially producing common communicative ground within pair families. Except for selection for facility in their use, no obvious reason is suggested for why or how these isolated gestures might have become subject to natural selection capable of creating language.

Yet the range of gestures needed was large in such close working relationships, perhaps 100s of gestures of hands, head, and arms as well as vocal and facial expressions would have become necessary. It is not hard to think of a long list: come, go, pick up, hide, run, quiet, look, listen, pull (root plants), carry, drink, water, infant come back, carry, I’ll carry, sit, cut, eat, suck, watch out, stop, keep back, give, no, yes. Perhaps they invented names for infants to call them and probably names for the animals, fruits, and vegetables they ate. Youngsters from different families probably joined together daily for play. Only seeing their full range of contexts and activities could we see the full range of communication they would need daily. Hominins needed all of these signals and many more to cooperate and help each other in close bonds, with perhaps a mobile youngster and an infant. Gestures seldom fell into logical sequences that needed grammar or syntax. The 100 emotions generated daily would already be expressed but perhaps modified in such close relationships.

Foraging together brought essential cooperation. Presumably this may have led to more support by males when increasing neonate head size led to the birth of altricial helpless infants when mothers again needed more help.

Tomasello (2014) saw the development of common ground and intentionality in social relationships as a first step toward what he describes as the common ground of groups. He makes no suggestions about which social relationships were involved. Few relationships would involve the demands for cooperation, integration, and common ground as would the relationships in pair families living separately within troops. The list above indicates some of the endless communication that had to go on within cooperating families. This is why an understanding of the societal structure of hominin troops becomes important.

For language to begin, separations were needed before new stories could be brought home. The separation could be fission–fusion or could be after hominins acquired a rotating clavicle allowing development of throwing skills (Gore, 2003) to be able to hunt; this allowed males to leave the foraging females, returning to camps in the afternoon after hunting. Separating males for hunting denied boy children knowledge of hunting models for play. Returning sub-groups of males and females had stories. Returning females would mimic the unusual behaviors observed while foraging. One mime might describe a female chasing and repeatedly beating a small mammal to death after missing with a single effective blow; females and males were amused. Perhaps it was a clumsy act by one female or the disorganized panic of discovering an infant had strayed that would be mimicked for amusement. This was sharing gossip, something all had enjoyed watching within troops for eons.

It is important to recognize this central activity of watching stories endlessly unfolding within primate troops. Daily everyone could watch a pestering youngster being weaned, youngsters playing “king of the castle,” a female approaching her male or even another female to groom. There are tiffs and reconciliations and sometimes fights. Tracking the behavior of others to understand its goal or meaning is quite general in many animals. Without it there could be no social facilitation and little socialization. How does tracking occur?

## TRACKING TO UNDERSTAND BEHAVIOR

Each moment of behavior is watched. The image of each movement was sensed, recognized with its function and probable interpretation, normally by comparison with an equivalent familiar relevant image in memory; how else could a movement or anything observed be recognized; (unless perhaps by a mirror neuron, Rizzolatti et al., 1996)? Mirror neurons have produced important behavior models and much controversy, e.g., Hickok (2013). Sensory input produces only images, visual, olfactory, or auditory. The movement images are thus sequenced and remembered/processed one by one until the goal of the behavior or interaction is understood. Goals are central to the creation and tracking of behavior. Frith (2007) wrote “To imitate someone, we watch their movements closely, but we don’t copy these movements. We use the movements to discover something in the mind of the person we are watching: the goal of their movement.” (Kindle Location 2275–2276). Modern children are well able to differentiate goals of behavior from the behavior itself, Bekkering et al. (2000). These studies were on people, but animal minds too seem to be designed for tracking behavior and determining its goals (Frith and Frith, 2012).

The goals of common behaviors would be quickly anticipated. Behaviors repeated as in social facilitation are familiar and easily anticipated. I suggest this tracking is little different from me listening to your words, referring the images of each word to memory for its recognition and identification, goals of images of phrases and sentences are held in sequence in working memory, bit by bit until I understand the sub-goals of the story you are telling me or anticipate its goal. More of this image tracking model below.

This sort of behavioral watching is part of socialization of young primates, a wonderful preparation for adult social and political life. No one would doubt that primates understand the social behavior and its goals occurring throughout their troop, e.g., Cheney and Seyfarth (2007). I suggest that this widespread animal skill was essential in the first great step to language, though it needed no language nor anything not already available in these primates.

## STORYTELLING WITHOUT LANGUAGE

Modern language has many descriptions and incredible complexity (Deacon, 1998). Yet in its beginnings it was simple and concerned with telling effective stories about people; it is this introduction to natural language and the process of its development that is the basis of this study.

I believe that models of language evolution still lack agreement, though support for the gesture origin is gradually increasing, perhaps encouraged by improved understanding of mirror neurons presented by Rizzolatti and Craighero (2004). The change is analyzed in detail by Corballis (2009). There remains no picture of why the first gestural steps might have attracted strong natural selection. One alternative approach seems more useful.

I am an ethologist and geneticist, not a linguist. With the support of linguist Thomas Sebeok, I presented a case for the evolution, not of language, but of storytelling by mime (McBride, 1968, 2000). Storytelling required no new behaviors, all the skills were available to hominins. It is a largely open system to handle any story by/about people. I suggested that after a spectacularly successful hunt, an alpha gave a play metasignal (Bateson, 1956) that stopped the hominin band rushing to eat and gave full attention to alpha. Alpha did not normally play. All the material of the hunt was present, hunters, weapons, and prey. Previous smaller mimes by females had given some preparation by their short gossipy play stories.

Alpha mimed the hunt story; he directed his hunters to tasks in their story, bringing them in. His watching troop had all observed young males playing hunting. Perhaps the mime included some abbreviating gestures like starting with; Go Stream, or Antelope Baby. The mime was easy to understand; the prey was there, dead. The feast would follow. The play metasignal merely changed the attention of watchers from the prey to the mime. The play metasignal communicates that what followed was “not real,” out of context, recursive, but they had no difficulty recognizing the story. What had been transmitted was something gestures alone could never do, present a *whole story,* a metaphor for an event. Every watcher understood how this hunt had been organized and why it was so special.

Mimes are not language. The proposal is that mimes came into being as a way of telling stories, *long before any possibility of language existed or was even anticipated*. Mime was a complete storytelling process well within the talents of the hominins in whose bands it occurred. These individuals had zero concept of language, but they could manage mimed stories and understand them. Mimes and their understanding required nothing that every hominin did not already have. The mime would have included every call and gesture used in the story; it was never a silent performance, nor was the hunt. Mimes took these hominins into stories of the past; the world of animals is mostly in the present. Miming whole stories gave an immediate evolutionary advantage, but first, an evolutionary perspective.

## PLAY AND LANGUAGE

The play metasignal has often been brought into language evolution studies. Thompson (2010) saw play as both a learned narrative subject to natural selection, but also as a protostory. He suggests storytelling makes personal episodic memories into group episodic memories. “Without the story, it is clear we would not be human” (p. 412). See also Coe et al. (2006). Episodic memories are the basis of experience, (Schwartz and Evans, 2001).

## ACQUIRING EXPERIENCE – STORYTELLING’S ROLE IN EVOLUTION

Throughout animal evolution, natural selection found various ways to ensure the production of reproductive adults. Sometimes enormous numbers of eggs and young were needed each generation to rear a few experienced adults to produce the next generation. Other youngsters evolved poisons that predators learned to avoid. Educating animals had always been costly but natural selection could only produce generalized adaptations, caution, excellent learning, memories, and sensory systems with rapid responses to danger. Perhaps reflection on dangerous episodic memories could involve a metacognition extracting specific lessons from the memorized experience, valuable for experienced adults; we know memorized experiences change, losing accuracy, but retaining lessons.

Early animals found meeting conspecifics was inevitable; learning adjustments to them was probably mainly by adopting spacing that prevented interfering with each other, perhaps on territories or sometimes associating closely when there were benefits from becoming gregarious. Youngsters joining adults would learn these expected spacing arrangements, being corrected when they came too close or crossed defended territorial borders, so that society itself became an early teacher, transmitting learned societal skills between generations.

In those species where family life evolved, two generations came together for extended periods allowing complexity to emerge within social relationships. These could now allow offspring to learn from observing the food and experiences of parents, especially a wide range of living experiences of many adults when in troops; this was a next step to the natural selection of adaptive learned behavior, transmitted between generations, early steps to culture.

Telling WHOLE stories gave every new generation access to 100s of living experiences second-hand from which to learn a thousand dangers specific to their lives within their own particular environment, especially hunting experiences for boys. Natural selection could never produce such diverse talents but it could add high attention, story learning, and the ability to access, and digest this experiential reservoir, becoming available when danger threatened. Stories contained more experiences than any youngster could expect to acquire personally in a lifetime. Telling whole stories was a single huge jump that potentially allowed youngsters to understand and prepare for experiences that could occur in their own future, the culmination of millions of years of natural selection to achieve experienced adults, cheaply, and effectively. Thereafter, evolving language simply made telling stories easier than full body miming. These youngsters grew to maturity well socialized, understanding, and prepared for everything that could happen to them in their lifetime. Natural selection had achieved this by creating a new evolutionary system, based on selection of learned adaptations, culture. The first human culture was certainly an experiential culture based on storytelling.

## LANGUAGE OR CONVERSATION?

What was also essential to the evolution of complex language from storytelling was the fact that telling stories required a double interaction;

(1) a primary animal social interaction between you and me, here and now and,
(2) a secondary interaction that contained the story.

It was this primary interaction engaging each other that provided the essential feedback, helping the teller to express the story better by allowing the receiver to express any needs for better explanations. Alpha had repeatedly looked at his audience to check that they were understanding his story. Feedback was the part of the communicative interaction we call non-verbal behavior, the ancient animal interaction between you and me, here and now. It was within these double interactions that language could slowly evolve, bit by bit from storytelling. Language could never have evolved its present complexity without the endless assistance that watchers and listeners gave to each other every day in every conversation. Linguists may be impressed by the extreme power and effectiveness of language, but it was never language that evolved; it was the conversation, *language’s evolutionary mechanism* – it still is!

Only the primary interaction could include the feedback essential to guide the slow improvement of ordinary language of evolving people in their contexts, bit by bit by learning the thousands of advances that became subject to learning and natural selection. Natural selection of behavior is probably always by “genetic assimilation,” Waddington’s (1953) term for Baldwin’s (1896) natural selection for facility in ability to acquire/learn new adaptive characters.

Language never evolved from an animal communicative system. It never evolved from gestures. Whole stories alone had the power of creating a vehicle that brought together the need for long sequences of structured behavior leading to the understanding of the goals of the behavior to be communicated. The evolution of language lies in the conversion of whole stories from mimes to a simpler system of signs and sounds which eventually completely replaced mimes and acquired the complexity it now has. The stories needed to transmit experiences to others, widening their experiences. Only thus would natural selection become involved and remain an important support during the long period while language evolved. Of course once an early language became possible, questions, and the contribution of receivers could move from non-verbal to language. Language in signing or sounds soon increased the range of topics possible, gossip, and of course discussions on moving camp or dangers from territorial neighbors. Planning hunts or foraging became possible. Language itself became functional socially and also subject to natural selection. That primary interaction always guided storyteller and recipients throughout the millennia that language evolved, very slowly. The path certainly included firstly mime including all the calls and gestures, then the transition to mainly gestures with some iconic pantomimes and calls, and finally to speech, still with lots of signs, and perhaps occasionally mimes. We still revert to mimes; how could I show you how awkwardly he limped without mime.

## NATURAL SELECTION FOR STORYTELLING

Why should storytelling be subject to strong natural selection? I suggest the following reasons.

- The first was the experience children gained from playing realistic hunts they had never observed, learning new skills from every story. Play became a real training for the day boys joined the adult hunters. Trained hunters brought more food to the troops, with fewer injuries.
- The second is the attention gained by skilled storytellers. Attention gives status and status brings many benefits. Good storytelling earned attention and respect independently of rank. Perhaps this was an early step from rank to status. Skilled storytellers might also have been subject to sexual selection.
- The third and most important was that those in the troop able to learn from the experiences of others now faced every new situation with relevant experiences greater than they alone could have acquired. To learn from others of a thousand specific dangers and successful reactions within their own environment increased their chances of escaping, to live to the next adventure. Stories were the culmination of natural selection’s great search to produce experienced adults without huge waste. Each sunset heralded story time. Every remembered adventure and escape could be shared. Over time, tribes accumulated large repertoires of stories, experiences, adding new ones, and recounting old well-loved ones passed from generation to generation. A treasure-trove was available to every wide-eyed child, cuddled close to its parents through the darkening mysterious evenings, soaking up every story. Have children changed? With language, stories create forums as different experiences are exchanged. Diamond (2014) has shown how such storytelling is ubiquitous today in New Guinea tribesmen.
- The final advantage lies in the ability of storytelling and teaching to transmit learned information, culture. A chimpanzee mother may “fish” for termites with a twig while the youngster watches intently. But she is emitting, not directing communication to her offspring, as a miming animal would in a double interaction. Teaching involves first gaining the attention of the other, then embedding a teaching interaction within the interpersonal interaction, the double interaction. The teacher demonstrates then follows each attempt of the pupil. Demonstrating can sometimes be miming. Animals sometimes teach but most learning is by observing. Hens in full display express fear or aggression fully while calling urgently to their chicks and bringing their attention to large spiders, teaching, a double interaction (McBride et al., 1969).

Storytelling forums are ubiquitous. At lunch with friends recently, one told of getting his car serviced at a large organization. The front tires had been ticked as checked but the friend found at home, that they were badly worn on the insides, illegal! Immediately a series of stories were told by others of experiences dealing with such organizations, a forum of information exchange emerged, all useful. Everyone has been in many such storytelling exchanges. We gain so much of our understanding of our culture from such forums, established for experience sharing by those early communicators.

## COMMUNICATING

Communication can be directed or emitted, deliberate or incidental, expected or unexpected. Scents deposited and birdsong (like modern radio and TV) are deliberate, emitted, and expected. They elicit no response. Every recipient selects the calls of its immediate neighbors to monitor, hears them at dawn. The dawn birdsong merely announces that society is as yesterday. If a call is missing, there is the unexpected information that a territory is vacant. All neighbors “hear” that missing call. They respond in their own interest. A non-territorial male becomes occupier of the vacant territory, now smaller than that of his predecessor; established neighbors have increased theirs. The society was restored by each individual acting in its own interests.

You walk through busy streets noticing nothing; all is emitted, incidental, anticipated, and expected information, Dennett’s (1992) “unconscious driving experience.” A bison tracks emitted incidental behavior of another settling for dust-bathing and joins it, social facilitation.

Alpha’s watchers had changed from watching emitted and incidental social and political stories daily to watching deliberate play stories, directed at them. Most story behavior in every primate troop or hominin band had always been emitted and incidental, observed by everyone. Storytelling brought these political and information events into interactions, as stories, deliberately, and directed. These were the stories of everyone else that eventually became social and political gossip, restoring the ancient pastime of all watchers, sharing everything happening in the troop. I suggest this is a simpler account than that of Dunbar (2010), though complementary. Grooming was retained but became limited, mostly within pair families. Some became grooming of children, more became cuddling or foreplay, privately in nests.

## STORYTELLING’S ROLE IN CONSCIOUSNESS

With natural selection incorporating storytelling into genetic constitutions, each step to language made great demands on individuals, particularly the need for immediate memory access to every detail of the episodic experiences in memory. How could alpha tell his story without controlled access to its memory and extended attention far beyond the brief high attention of the Orienting Response (OR). We have retained the OR, but also taken its high attention, mental access, and attention to memories and extended them, essentially indefinitely.

We believe animals are mostly aware, living in an anticipated present and dealing with arriving sensory input, Dennett’s “unconscious driving experience” that we have acquired from our ancestors. We too can walk or drive from A to B remembering nothing of the trip. It may be through busy streets, but we are aware, not conscious. The aware state is still busy, comparing all sensory image input with the expected images in memory. If all is as expected, the comparison remains only in working memory and is forgotten, clutter. Only an OR to detecting something unexpected would bring the individual to attention, detecting an image not present in the image maps and social models in memory. In the OR, a “consciousness” brings access and attention to other relevant recalled memories to make decisions, to flee, seek more information or ignore, ancient responses, widespread in animals.

Access to physical and social memories in the aware state and the OR are central to expected anticipated animal and human life. Each sensory image input appears to be the key that specifies which specific images should be recalled from memory for comparison, an ongoing, and endless process in awareness; better control of access to memories was probably seldom needed outside the OR and possible meta-cognition in animals. Detecting any change or recognition is impossible without comparison of two images. Monitoring for absence of change provides the stability of animal social and environmental living. They live in expected, anticipated worlds, or within the “social norms” of sociologists. The OR alerts to any change in physical and social environment; it is a moment when the individual needs more information and the ability to make decisions. Animals have been making decisions for millions of years. The moments of OR are probably the nearest animals come to consciousness, and probably the OR provided that base equipment from which natural selection shaped human consciousness (McBride, 2012). In ORs, improved access to memories was important if previous experiences or story experiences were to be used. Telling stories also needed far better control over memory recall, unnecessary in normal animal awareness. It needed extended consciousness, extending the OR considerably.

Temporary memory is important in tracking the behavior of others, bit by bit until each sentence goal of the behavior had been understood, and again until the interaction sequence or story was completed. Later we will consider an alternative that involves less dependence on memory in this process. Yet any animal needed to track behavior to understand the goals of behavior and interactions all around them in troops, a basis of socialization. It became more demanding for mimes out of context; presenting contexts would always have been the difficult part of mimes.

Searching in memories has always been a demand for animal minds. The environmental maps of familiar paths in arboreal primates were three dimensional. Deciding to go to drink from any part of the range or a sudden need to flee meant choosing a path, mentally moving a self-image freely through alternative memory maps to decide on a plan. Is this any different from you remembering the house you grew up in and moving your attention through each room in your memorized maps. Is it different from moving your attention through the complications of language evolution? Is this thinking? Does it sometimes have a spatial origin? To move through the multidimensional experience memory to choose a linear path for a story again makes special demands on memory access.

While the evolution of conscious thinking has many threads, the demands of storytelling have certainly contributed. Every child needed to remember each story, often moving through its images repeatedly in extended consciousness to make sense and reality of it; with elementary language, asking questions about it. Consciousness was emerging as ready access to those memories improved. In this way, the stories could be turned into second-hand image experiences in memory, to be worked through repeatedly, available when needed.

Understanding experiences, episodic memories remains a puzzle of great importance. Merely remembering some episode does not create an experienced adult. Always many parts of the experience are irrelevant while some features need examination if learning from experience is to be meaningful. We know that any dramatic experience in people is likely to come to the fore in memory, often repeatedly. We know that we seek to locate the essential features of the memory, and the feeling of satisfaction that follows this understanding. The memory now becomes settled, less likely to pop out again. The memory itself will have changed. We have this skill in digesting episodic memories: do animals? Clearly it evolved in us from something in animals and animals have always faced these similar problems.

Perhaps some understanding of experiences might have occurred by mind wandering (Corballis, 2014) into experiential memories. We have such mind wandering: do other species? Memories of experiences have always needed “digestion” to become functional. What could minds do with such experiences except move through the image sequences, perhaps repeatedly. In ORs, some images would elicit recall of other relevant memories; this is normal in ORs where they are matched, compared, and perhaps differences discovered. We know that becoming an experienced adult is important if benefits are to accrue. Some “digestive” process is required for learning from experiences to occur. Is this only in us or have we inherited such an important process? Where would it have been more important than in youngsters digesting the many stories they were hearing? All involved well extended conscious attention to experiences or story image sequences.

Giving high and extended attention to the stories of individuals could hardly have failed to elicit prosocial and empathic feelings toward those telling exciting stories, Gaesser (2012). Like any favorable mutual experience, sharing storytelling would contribute to the feelings of solidarity within the troop.

Those moments of high attention and decision making in the OR are obvious precursors from which natural selection could build a human consciousness. ORs are extended in time in many other situations. The OR is normally short, but in exploration and hunts this high attention is extended presumably with access to memories still relevant to the situation. An animal moving to a new area explores, probably comparing what it sees by searching for equivalent images in memory, particularly dangerous images. When this over, it has normally “chosen” places for resting and sleep, for drinking and the paths between them. Decisions were made – on experience? Learning is central to the OR, also to exploration and hunting. Pavlov’s (1927) OR, was developed as important by another Russian scientist, Sokolov (1963) who recognized that the OR was a significant behavior; since then, 100s of studies have been made, mostly on people and caged animals, but not by ethologists who see ORs daily in natural situations. The OR has not been related to exploration or hunting.

## SELF PRESENTATION

The self has always been central to animal evolution. Animals were concerned with and learned only what was relevant to self, stored in maps, and memories. *Their environment is that part of surroundings that is in memory, monitored, and eliciting an OR should any difference occur between what is sensed and what is in maps/models.* Environment is not surroundings.

Self took a new role once storytelling emerged. Telling a story of self included using the self as a “third person” in the mime or signed story. Self could be presented, favorably? What self-image was to be presented? Did this bolster the expansion of self-image in early human minds? Perhaps achieving high status or winning a higher rank had always demanded some self-presentation. Animals displaying on their territory had always presented self – with some “consciousness”? Certainly our recent move into anonymous cities meant that strangers could only judge individuals by what they could see; this new self-presentation has created enormous industries. Every individual today grows expecting to present self, urged by mothers to “look your best” when going outside among strangers.

## MIME LIMITATIONS

Mime could tell only some stories and ask no questions. It was a clumsy way of storytelling. When language improved, questions bubbled out and a thousand types of stories emerged, of battles, moving camps, important stories that could never be mimed. Youngsters could be taught how to throw a spear or build one. Teaching blossoms within double interactions as opposed to learning by observation. Storytelling began as metaphors of real events. More metaphors and similes were essential in bringing complex objects, categories, places, contexts, and events info stories, expanding vocabularies. Every step to signing and language reduced the limitations of miming, with less effort!

## CULTURE

I have argued that elementary learned and selected “cultures” evolved first within and created primitive animal societies, were later enhanced enormously within family life, especially in troops, and exploded with storytelling. The first significant human culture to emerge was an experiential culture, a highly functional culture gained by storytelling, expanding with use of language.

The double interaction brought teaching into any interaction, demonstrating/miming. Every new demand for innovation in this communicative system put pressures on the language part of the transition. Asking questions must have fostered the transition to language; questions also demanded further extension of the high attention of the OR. I am only aware of the thousand steps from mime to signs/sounds and thence to a modern spoken language; only a linguist could unravel them. I suggest that it was this primary interaction that was the source of endless feedback that provided the thousand steps that were subject to natural selection in the gradual “Baldwinian genetic assimilation” of human language skills.

Emotion is part of all storytelling and language, expressed constantly in momentary movements of the face and body. There could be no story without the expression of the feelings of the players, signers, or speakers. Stories were mostly about people. Expressing these feelings has obviously been selected naturally. The modern human face has genetically assimilated the capacity for complete expression of every momentary feeling evoked by the story or the speaker. The use of the face to express these feelings was part of effective human communicating; without it there could be little of the mutuality and joint intentionality emphasized by Tomasello (2010). Darwin (1872) brought emotional expression to general attention and especially Ekman (1973) showed how effectively the individual muscles of our faces have evolved to express our feelings, moment by moment, providing feedback.

## MIMES TO LANGUAGE, THE EVOLUTIONARY TRANSITION

I have proposed that storytelling was the dramatic step toward language that required nothing not available to the animal minds of hominins. Lieberman (1988) proposed that grammar already existed in behavior, emphasizing the hierarchical nature of behavior, and language. Fodor (1975) saw representational systems with symbols and their manipulation as central to thought, “a language of thought.” But he thinks of thought as we do, as people. Animals “think” in many OR situations; they presumably use images; we know of no alternative. Perhaps some are equivalent to Fodor’s symbols. Animal minds are filled with images; it is hard to believe that behavior goals do not exist in images. They have visual images of everything in life in their memories; their world is an image world. Animal thinking must rely on these images in ORs. They are of scenes but also with categories, water, monkeys, trees and branches, rain, wind and sunshine or cold, images they see or feel. Animals set goals for any decision to make behavior, how else but by images, of what, the behavior or its goal? The repertoire of gestures, calls or facial expressions suggested as essential for cooperating hominin pair families would have become the images for these goals. Perhaps it was the image model of thinking that was also the behavior planning language; if so they were together changed into human languages.

Thinking in ORs includes deciding and planning the next behavior. Decision making is ancient, long preceding any logical thinking processes, probably normally relying on feelings in a situation, perhaps remembering images or feelings of similar situations; these recalls and comparisons gave a conservative security. Some decisions are delayed; more information is needed, especially in social situations. Planning next behavior can be demanding.

Lieberman (1988) drew attention to language and behavior similarities, both organized hierarchically. However, I was introduced to a different behavior planning and hierarchical structure in an exciting lecture by Karl Pribram (Miller et al., 1960). Perhaps there is a language of behavior implied by them, hierarchically organized to deal with the incredible complexity of the behavior planning and generating process. Yet the picture I have from years of worrying about their model is that *there are two languages, one that deals with behavior goals in planning in the mind and another that deals with behavior generation,* organized unconsciously by the brain, a *“planning language” and a “programming language.”* Let me enlarge.

Our primate decides to drink. Perhaps the plan is “get up and go to place A. It is always dangerous there so look around. Then on to place B where a long jump between trees is needed and on to place C overlooking the stream. Then watch and if safe, descend to water. Look around carefully. Drink, look around. Get back into trees.” This is a series of major intermediate goal images that may flash through the mind, in a troop, all of the minds. Clearly at each stage of the operation, the next sub-goal will flash into mind, but the journey continues; each new goal is followed by the next plan, anticipated momentarily. The whole plan comprises lots of ever smaller goals, each monitored as the journey proceeds.

The behavior generating program for the goals is more complex; a hierarchy of 100s of muscles must be pulled in perfect timing to achieve every single movement in the program for the first goal to move self up and turn to the right direction. Moving the body then demands that every limb, hand, finger, foot, and toe muscle pulls must be organized to achieve each tiny sub-goal, untold programmed muscle pulls, each exquisitely timed, controlled, and monitored throughout to deal with many more smaller goals, for jumping between and running along branches to place A. All is familiar, with well-established sub-programs.

I decide to make a cup of coffee, a plan of standing up, walking across the room, turning through the kitchen door, filling the jug, and heating it, etc., so many intermediate goals. My brain has the bigger job to program the muscles for throwing my heavy head forward, then arranging muscles for my legs to catch my body weight for standing up, then those legs need the pulling of many muscles, perfectly timed to walk, a familiar action. I locate the jug and another programming takes over to organize the hands to fit the jug and cup, well described by Frith (2007). The unconscious program my brain produces involves thousands of muscle pulls of controlled force, superbly timed, *and monitored*; it is the incredible flawless programmed and monitored job that occurs in every activity, introduced to me by Karl Pribram.

The planning language deals with a hierarchy of goals. *It is the simplified language of goals we use in discussing behavior.* It is the planning language we were forced to use throughout the evolution of language. Yes, extracting a goal plan from the experiential memory of the hunt was central to generate the story mime. I suggest that to move from the mime, our ancestors had to use the simpler language of behavior goal planning. No story could be told if the hominins were forced to tell the thousands of movements they saw, though they had no alternative when miming the story! Goals are central to the condensation of behavior stories. Plans of goals are the same stories before they are programmed into detailed muscle pulling behavior. They are the story created in minds, consciously from memory. They were inevitably the second step to language after the value of whole mimed stories had been established by natural selection, the first step.

Stories were the vehicle that created the demands for simplifying as much behavior as possible to signs, calls or gestures that could represent goals in any story. Any mimed story could start with known signs for, “go stream” and end with, “come back.” An action could be to mime the spearing of antelope infant or to gesture, “spear baby.” The story always provided the vehicle in which many of its small goals could be inserted into the mimes as pantomimes or by any gestures known within the troop. And every youngster benefitted from easy understanding of the mixed mime/gesture story.

These hominins had acquired gestures for 100s of communicative exchanges used daily in cooperating family units; the mental representation of each gesture became the mental goals for the behavior elicited by them. The behaviors of mimes already depended on planning goals and images, some used to produce that first mime. Many, eventually all goals became gestures or sounds. Simplification converted mental goals into gestures or calls. The simplifying step from whole mimes was to use any goal images which already had gesture or call equivalents. If these were understood throughout the troop, they could substitute for sections of, or eventually the whole mime. And families had already acquired very many short cuts in gestures and calls. When any change was needed, these gesture/calls were what appeared mentally as the action was performed. These represented ordinary activities of people, available for substitution for goals of the mime, simplifying it, yet still understood by everyone. Obviously more were needed to create new goal gestures, probably iconic or onomatopoeic for easy acceptance.

Language evolution constituted the transition from mime stories to the “language” of planning goals to tell the stories. It was another use for the existing behavior planning process, but now used quite differently as a complete communicative device. Behavior planning goals remained subject to all the rules and organization that had always applied to behavior; it was itself just a behavior generated as was every behavior but it made storytelling very much easier. It also made the transition from mime to a potential language straightforward and probably inevitable for a species already familiar with communicating by a wide range of gestures/calls.

I have emphasized the dual role of gestures and calls to counter the assumption that the transition was completely to signs then to speech. There had never been any need for silence while communicating around the camp.

## MECHANISMS

Animals and people have this generating process converting goals into detailed muscle behavior. Tracking behavior involves converting detailed observed behavior movements into goals, a reverse of the generating process. Would natural selection create two different complex mechanisms, or a way to use the same generating process for the demands of the two directions of generating and tracking? I suggest there is one mechanism. The double functions of the mirror neurons strongly suggests their roles and perhaps other similar components in both generation and tracking, though how big a role remains to be discovered. The system would need to involve learning rather than creating separate mirror neurons for every movement, sign, and phoneme as these evolved complexity.

We know we have a behavior generating process that turns goals into functional behavior, including a mimed story. We know we have a speech system that turns mental goals into verbal stories. We know that watching the mime can convert the behavior into goals and understanding. We know that listening to the same story, bit by bit yields the goals, and understanding of the story. We know that there exist mirror neurons that could possibly be used both to produce and follow a story. We are unsure of how ubiquitous are these mirror neurons or whether they could handle both learned words and signing movements, but we know that words can serve both speech and listening, as can mirror neurons and perhaps more general mirror equivalents, including learned equivalents.

Thus tracking signed or word language, each goal sign or sound is sensed, recognized along with its meaning and implications and transmitted to the generating mechanism to recover the sequence of hierarchical goals until the story is recovered. The generating language is the only mechanism we know is capable of turning goals into behavior and behavior into goals, including story understanding. *We have taken the planning language and made it our language, for generating and tracking behavior.* It held the possibility of all the subtlety of sub-planning and sub-sub-planning. With experience, everyone could understand it as easily as watching the mime. This was the language that was easy to move to signs/calls; hominins were already using a planning function depending on common understanding throughout the troop. They already had facility in recognizing the many signs/sounds within their common contextual understanding. Using signs and calls in the behavior planning language was not a large step in interactions.

If there is a universal syntax, it involves both generating and tracking in behavior and languages, in signing and words, for example in generating fights and tracking every detail of the opponent’s movements for goals needing responses. Certainly the move to words, phonemes, and morphemes demanded the same sort of intermediate goal recognition required for bit by bit tracking as does signing and the watching of troop social behavior. I have often wondered how I remember the many words in a long sentence to arrive at or anticipate the sentence goal. But I suggest that memory alone is not involved. The reverse behavior generating mechanism is actively assembling this goal understanding. *Memory alone could never do this.*

With detailed programming language, one mistake in the syntax of the plan would destroy the whole behavior. Instead of getting up to make a cup of coffee, one could fall flat on one’s face. Such an efficient behavior planning and programming skill/language must have evolved early in animal evolution; one does not see animals failing to reach goals or falling over.

The incredible effectiveness of behavior generating that allows every animal to plan goals transmitted into activities involving thousands of muscle pulls, all perfectly timed to achieve the goal without ever a mistake is suggestive of an inbuilt “universal syntax” for behavior and language generation. If this existed, it would be hard to deny that such a “universal syntax” suggested by Chomsky (1957) became involved in our languages though he saw it as specific to language – he was a linguist. Natural selection is unlikely to have evolved a second such similar powerful mechanism. It would be this same system that generates the movements of mouth, tongue and breathing in response to the flow of word images being called by mind and brain.

Is there an alternative model to a universal syntax? There are equally ancient monitoring systems throughout all behavior and language generating systems allowing rapid corrections in the generating processes. A learned monitoring program is possible that stops the generation of muscle instruction sequences that have not worked in the past, keeping to familiar, effective, practiced or cultural sequences while carefully controlling and monitoring new ones. Monitoring includes many functions, reaching goals, feedback from others, from cultural expectations. In all cases, there is monitoring of *all behavior* as it occurs. Tomasello (2003) has suggested a “user based” developmental model of language acquisition, an effective alternative, though I saw a systems approach as an effective alternative to the acquisition process in infants and children. Infants would, like us build on existing expectations, observing, and responding to each change they observe around them, with age and previous steps always part of the feedback processes. How would we choose between these models?

Behavior or story plans are usually linear, one dimensional. The activity generated is likely to emerge as multidimensional, as in planning that drink. The plan is in the mind while the behavioral generation for the program is unconscious in the brain and is not linear. It was this double behavior planning and programming that produced that first mime. It was certainly adaptable enough to transfer from memory a goal plan in “planning language” to include every critical goal and every call and sign used within the story. The hunt had been many dimensional with many smaller sequences, but the hunters each chose a single dimensional story plan to be shaped into the muscle details of his mime. Their behavioral planning skills gave them freedom to choose what to mime and what to ignore. All the mind required was the ability to use memory to plan goal elements of the hunt, probably a simple linear story, a goal story. Memory would have aided the generation of a realistic acting storytelling, ignoring the images of the context.

This questions the format of episodic memories. How much is in behavior planning language and how much in full image memories of the behavior observed. Clearly the episodic scenes observed were memorized as full images. Yet when episodic memories are examined for understanding, this requires their transition to goal planning language, probably an efficient change. Transitions to a goal language may assist analysis if this occurs. Full images are clearly stored as memories, but any mental examination uses the simple language of goals.

When miming, signing or speaking a story from memory, the mind chooses only one story plan, but it is the detailed programs for the brain that must now differ. This step to brain programs now depends *only* on *learned* mechanics to send to the behavior programming algorithm. Whole body plans for mimes used the ancient skills; signing demanded *learned* skills of programming arm, hand, facial, and vocal muscles to produce a quite different signed behavioral version of the same story. Modern speech involves many muscles performing quite different learned sound producing actions from telling a story by mime or signs. Yet the same story goal image sequence in memory and choice of a story plan is not changed but chooses images of words representing sequential goals, and a learned program for the thousands of individual facial, mouth, and breathing muscles is elicited, and monitored. The movements of speech may be learned, but the ability to create this range of sounds was genetically assimilated.

The transition of planning goals to signs and words was certainly a big learning and genetic assimilation process. The planning process needed to change as signs were learned to replace image goals and sub-goals, creating firstly sign programs without altering the plan. A complete change of the muscles now had to be learned to present the story in signing and speaking. Once learned, (and the ability to make the conversion presumably genetically assimilated) the same behavior planning process was appropriate, but quite different sets of movement instructions were needed to generate the same story. We still learn these different skills; thousands annually learn facility in signing and speaking different languages.

I suggest that storytelling in mime, signing, and speech and also episodic memories involve the same language of behavior goal planning, with human language now becoming a more efficient thought process in goal-words than images. It is probably the same language of thought in images of Fodor, now with words, and for the deaf, signs replacing goal images. We needed access to different learned sets of muscle movements for signing and speaking. And each of us can learn any of these, signing and talking any language.

Was it likely that a completely new mechanism would have evolved to produce each step to speech? Natural selection modifies (exapts) existing characters where possible, and very efficient behavioral mechanisms already existed. In both behavioral and speech planning, any syntax seems likely to have origins in the languages of behavior planning/generation. In miming, signing or speech, it is a language of goal planning that is converted into the learned muscles generating each format.

There are always many possible ways of generating any behavior or telling any story. The mind may choose one or let the brain make the choice as well as create the plan that will also involve a choice of specific action components. This would be as true of language as any other behavior.

Stories are multidimensional with many things happening simultaneously yet the story, planned, signed or spoken is generated linearly. No two people would tell the same story of any experience. *Minds evolved to plan and select linear goal stories of any event from memory as they chose linear plans for any behavior*. Complex episodes needed to be coded only in behavior planning language. Consider how we think linearly of making that cup of coffee, quite different from the plan the brain must produce to push every detail of a body image through familiar surroundings. With these evolved incredible skills of hierarchical behavior generating to make a cup of coffee, we can plan the many dimensional construction of a skyscraper or an Olympic games!

Summarizing, the detailed movement image of the story has become irrelevant in the transmission of stories. The simplified word or signed image of each goal of the story satisfies the needs of the communication. It is understood in enough detail, with extra available by questions when needed, without the need for a full imaged movement picture of the story entering memory. Our skills in reading are another learned process. Clearly this more efficient processing of information was part of a recent cultural evolutionary process. We retain the ability to track behavior as in movies and the incidental stories we still see daily.

## WHEN?

Questions have been raised about how recent was the evolution of human language. There are two parts to the question. One is the time needed for storytelling to be converted to speech with the whole final process genetically assimilated throughout the species. This occurred in small semi-isolated hunting-gathering bands with regular exchange of individuals with each advance occurring probably in a different band, spreading between neighboring to distant bands.

Wright (1931), the great American geneticist, presented the case for ideal conditions for rapid natural selection – a large population divided into small semi-isolated subgroups; he could have been describing the hominin population throughout most of its evolution. The natural language evolution from storytelling planning to speech fits his model, using both within and between band genetic variance. With such a spatial subdivision the evolution would not have been rapid.

The second is the time needed to spread modern speech throughout the whole human population, omitting no one. On this, we know that our species acquired genes from both Neanderthals and Denisovans. Thus the single spread of one source of ancestors “out of Africa” is no longer enough to account for the spread of language from a single source unless these species all had language. Upsetting an evolving language genotype by crossing with a non-linguistic species would not have been a minor genetic event, but one quickly eliminated by natural selection unless there were some other functional advantages to be retained from the cross. Even then, the spread of functional genetic material and selection against the harmful effects on genetic assimilation of communication handicaps would have been complex and in competition with the genotypes of bands free of outcrossing. Selection within and between bands would be difficult to estimate. I suggest that language evolution was probably complete before the separation of the African from the Neanderthal and other later contributors to our modern genome. The spread of genes from these crosses continues without effect on language. Did it always?

The effectiveness of selection depends on various factors, the characters, and their number, their heritabilities and correlations, the intensity of selection of each character in each band, the band size, and the amount of exchange between bands. We assume that hominins lived in small bands with some contacts between bands and that these bands split when they grew large. Without splitting, there could be no accounting for the millions of bands that spread throughout the world at least twice. Like chimps, it would probably have been the females that moved between bands, with the males responsible for maintenance of territory. Language evolution was probably complete in both Neanderthals and *Homo sapiens* when they crossbred while the latter reconquered the earth.

## Conflict of Interest Statement

The author declares that the research was conducted in the absence of any commercial or financial relationships that could be construed as a potential conflict of interest.

## References

[B1] Baldwin J. M. (1896). A new factor in evolution. *Am. Nat.* 30 441–451 10.1086/276408

[B2] Bateson G. (1956). “The message ‘This is play’,” in *Group Processes: Transactions of the Second Conference* ed. Schaffner B. (New York: Josiah Macy Foundation), 145–242

[B3] Bekkering H. Wohlschlager A. Gattis M. (2000). Imitation of gestures in children is goal-directed. *Q. J. Exp. Psychol. A* 53 153–164 10.1080/713755872 10718068

[B4] Cheney D. L. Seyfarth R. M. (2007). *Baboon Metaphysics: The Evolution of a Social Mind*. Chicago: Chicago University Press 10.7208/chicago/9780226102429.001.0001

[B5] Chomsky N. (1957). *Syntactic Structures.* The Hague: Mouton & Co

[B6] Coe K. Aitken N. E. Palmer C. (2006). Once upon a time: ancestors and the evolutionary significance of stories. *Anthropol. Forum* 16 21–40 10.1080/00664670600572421

[B7] Corballis M. C. (2003). *From Hand to Mouth: The Origins of Language.* Princeton: Princeton University Press

[B8] Corballis M. C. (2009). The evolution of language. *Ann. N. Y. Acad. Sci.* 1156 19–43 10.1111/j.1749-6632.2009.04423.x 19338501

[B9] Corballis M. C. (2014). Wandering tales: evolutionary origins of mental time travel and language. *Front. Psychol.* 4:485 10.3389/fpsyg.2013.00485 PMC3725404 23908641

[B10] Darwin C. (1872). *The Expression of the Emotions in Man and Animals.* London: John Murray

[B11] Deacon T. W. (1998). *Symbolic Species: The Co-Evolution of Language and The Brain.* New York: W. W. Norton & Co

[B12] Dennett D. C. (1992). *Consciousness Explained.* Boston, MA: Back Bay Books

[B13] Diamond J. (1991). *The Rise and Fall of the Third Chimpanzee.* London: Vintage

[B14] Diamond J. (2014). *The World Until Yesterday: What We Can Learn From Traditional Societies*. London: Penguin Group, Kindle edition

[B15] Dunbar R. (2010). *Grooming, Gossip and the Evolution of Language.* London: Faber and Faber, Kindle Edition

[B16] Ekman P. (ed.) (1973). *Darwin and Facial Expression.* New York: Academic Press

[B17] Fodor J. A. (1975). *The Language of Thought.* New York: Thomas Crowell

[B18] Frith C. D. (2007). *Making Up the Mind: How the Brain Creates Our Mental World.* London: Blackwell Publishing, Kindle edition

[B19] Frith C. D. Frith U. (2012). Mechanisms of social cognition. *Annu. Rev. Psychol.* 63 287–313 10.1146/annurev-psych-120710-100449 21838544

[B20] Gaesser B. (2012). Constructing memory, imagination and empathy: a cognitive neuroscience perspective. *Front. Psychol.* 3:576 10.3389/fpsyg.2012.00576 PMC3579581 23440064

[B21] Gore R. (2003). The rise of mammals. *Natl. Geog.* 2–37

[B22] Hediger H. (1962). “The evolution of territorial behaviour,” in *The Social Life of Early Man* ed. Washburn S. L. (London: Methuen), 17–33

[B23] Hickok G. (2013). Do mirror neurons subserve action understanding? *Neurosci. Lett*. 540 56–58 10.1016/j.neulet.2012.11.001 23147121 PMC3596437

[B24] Hrdy S. B. (2009). *Mothers and Others: The Evolutionary Origins of Mutual Understanding*. Cambridge, MA: Harvard University Press, 422

[B25] Lieberman P. (1988). On the evolution of human syntactic ability: its pre-adaptive base – motor control and speech. *Hum. Evol.* 3 3–18 10.1007/BF02436588

[B26] McBride G. (1968). On the evolution of human language. *Soc. Sci. Inform.* 7 81–85 10.1177/053901846800700507

[B27] McBride G. (2000). *The Genesis Chronicles: The Evolution of Humankind.* Sydney: Allen & Unwin

[B28] McBride G. (2012). Ethology, evolution, mind and consciousness. *J. Conscious. Explor. Res.* 3 830–840

[B29] McBride G. Parer I. P. Foenander F. (1969). The social organisation and behaviour of the feral domestic fowl. *Anim. Behav. Monogr.* 2 127–181 10.1016/S0066-1856(69)80003-8

[B30] Miller G. A. Galanter E. Pribram K. H. (1960). *Plans and the Structure of Behaviour.* New York: Rinehart & Winston

[B31] Pavlov I. P. (1927). *Conditioned Reflexes.* Oxford: Oxford University Press

[B32] Pinker S. Bloom P. (1990). Natural language and natural selection. *Behav. Brain Sci.* 13 707–784 10.1017/S0140525X00081061

[B33] Rizzolatti G. Craighero L. (2004). The mirror-neuron system. *Annu. Rev. Neurosci.* 27 169–192 10.1146/annurev.neuro.27.070203.144230 15217330

[B34] Rizzolatti G. Fadiga L. Gallese V. Fogassi L. (1996). Premotor cortex and the recognition of motor actions. *Cogn. Brain Res.* 3 131–141 10.1016/0926-6410(95)00038-0 8713554

[B35] Schwartz B. L. Evans S. (2001). Review: episodic memory in primates. *Am. J. Primatol.* 55 71–85 10.1002/ajp.1041 11668526

[B36] Sokolov E. N. (1963). Higher nervous functions: the orienting response. *Annu. Rev. Physiol.* 25 505–550 10.1146/annurev.ph.25.030163.002553 13977960

[B37] Thompson T. A. K. (2010). The ape that captured time: folklore, narrative and the human-animal divide. *West. Folk.* 69 395–420

[B38] Tomasello M. (2003). *Constructing a Language: A Usage Based Theory of Language Acquisition.* Cambridge, MA: Harvard University Press

[B39] Tomasello M. (2010). *The Origins of Human Communication.* Cambridge, MA: MIT Press, Kindle edition

[B40] Tomasello M. (2014). *Natural History of Human Thinking.* Cambridge, MA: Harvard University Press, Kindle edition

[B41] Waddington C. H. (1953). The “Baldwin Effect,” genetic assimilation and “homeostasis.” *Evolution* 7 386–387 10.2307/2405346

[B42] Wheeler P. E. (1988). Stand tall to stay cool. *New Sci.* 118 62–65

[B43] Wheeler P. E. (1991). The influence of bipedalism on the energy and water budgets of early hominids. *J. Hum. Evol.* 21 107–136 10.1016/0047-2484(91)90002-D

[B44] White T. D. Asfaw B. Bevene Y. Haile-Selassie Y. Lovejoy C. O. Suwa G. (2009). *Ardipithecus* ramidus and the paleobiology of early hominids. *Science* 326 75–86 10.1126/science.1175802 19810190

[B45] Wright S. (1931). Evolution in mendelian populations. *Genetics* 16 97–159 17246615 10.1093/genetics/16.2.97 PMC1201091

